# Non-coding RNAs as epigenetic regulator of glioma stem-like cell differentiation

**DOI:** 10.3389/fgene.2014.00014

**Published:** 2014-02-03

**Authors:** Keisuke Katsushima, Yutaka Kondo

**Affiliations:** Division of Epigenomics, Aichi Cancer Center Research Institute, NagoyaJapan

**Keywords:** epigenetics, glioma, cancer stem cells, long non-coding RNA, micro RNA

## Abstract

Glioblastomas show heterogeneous histological features. These distinct phenotypic states are thought to be associated with the presence of glioma stem cells (GSCs), which are highly tumorigenic and self-renewing sub-population of tumor cells that have different functional characteristics. Differentiation of GSCs may be regulated by multi-tiered epigenetic mechanisms that orchestrate the expression of thousands of genes. One such regulatory mechanism involves functional non-coding RNAs (ncRNAs), such as microRNAs (miRNAs); a large number of ncRNAs have been identified and shown to regulate the expression of genes associated with cell differentiation programs. Given the roles of miRNAs in cell differentiation, it is possible they are involved in the regulation of gene expression networks in GSCs that are important for the maintenance of the pluripotent state and for directing differentiation. Here, we review recent findings on ncRNAs associated with GSC differentiation and discuss how these ncRNAs contribute to the establishment of tissue heterogeneity during glioblastoma tumor formation.

## INTRODUCTION

Gliomas are the most common type of malignant primary brain tumor with an incidence of ~5 cases per 100,000 persons (Wen and Kesari, 2008). Glioblastoma multiforme (GBM) is the highest grade glioma (grade 4). Despite advances in treatment using combinations of surgery, radiotherapy, and chemotherapy, GBM confers an average life expectancy of around 14 months from diagnosis (Wen and Kesari, 2008). Accumulating evidence indicates that the presence of a subset of cells with the potential to initiate and maintain growth of gliomas might be crucial for their resistance to conventional therapies (Hadjipanayis and Van Meir, 2009). These cells are designated as glioma stem cells (GSCs; Galli et al., 2004; Singh et al., 2004; Lee et al., 2006; Penuelas et al., 2009; Mazzoleni et al., 2010). GSCs and normal neural stem cells appear to share common features including self-renewal and the capability of differentiating into multiple lineages. Intriguingly, recent studies revealed that in addition to GSCs differentiating into non-GSCs, the reverse process might also occur (Gupta et al., 2011; Natsume et al., 2013). This phenotypic plasticity between the GSC and non-GSC states may be regulated by signals within the tumor microenvironment.

Microenvironmental signals, such as sonic Hedgehog (SHH), Wnt, and Notch, have been shown to regulate the properties of cancer stem cells (Reya and Clevers, 2005; Fan et al., 2010; Takebe et al., 2011). SHH has a critical role in the maintenance of GSCs by regulating so-called “stemness” genes and has also been found to be activated in many high-grade gliomas (Clement et al., 2007; Takezaki et al., 2011). The Wnt/β-catenin pathway has been implicated in the role of GSCs in gliomagenesis through tumor proliferation and invasion (Nager et al., 2012). Notch signaling has been shown to promote GSC self-renewal and to suppress GSC differentiation (Shih and Holland, 2006; Fan et al., 2010; Hu et al., 2011). Genes in the receptor tyrosine kinase (RTK) family mediate several oncogenic growth factor pathways, such as epidermal growth factor receptor (EGFR) and platelet-derived growth factor receptor (PDGFR), that have been linked to malignancy, angiogenesis, self-renewal, and multipotency. Recently, it was shown that constitutively activated EGFRvIII expression and loss of the phosphatase and tensin (PTEN) protein in murine neural stem cells results in the formation of glial tumors (Li et al., 2009a). PDGF overexpression has also been implicated in gliomagenesis, and PDGFs can inhibit glial cell differentiation (Fomchenko and Holland, 2007).

Recent advanced technology to identify non-coding RNAs using microarrays or next generation sequencing technologies provide extraordinary abundance of novel data in genome wide-scale and revealed deeper insights into the biology of non-cording RNAs (ncRNAs). More than 90% of the human genome appears to be transcribed and transcription is not limited to protein-coding regions (Birney et al., 2007). Some ncRNAs may play key regulatory and functional roles. Indeed, significant numbers of ncRNAs, such as microRNAs (miRNAs, miRs) and long non-coding RNAs (lncRNAs), are regulated during development in a cell-type specific manner, and are associated with multiple cell functions (Kapranov et al., 2007). miRNAs are the short non-coding endogenous RNAs that post-transcriptionally regulate the expression of a large number of genes (Bartel, 2004). miRNAs play important roles in a wide variety of physiological and pathological processes including tumor formation. Aberrant expression of miRNA can induce tumor suppression or can have an oncogenic effect resulting in tumor formation (Medina and Slack, 2008; Gangaraju and Lin, 2009). lncRNAs are functional ncRNAs that are potentially key regulators not only of cellular differentiation and proliferation, but may also have tumor suppressive or oncogenic functions in many types of cancer (Esteller, 2011; Wapinski and Chang, 2011; Hu et al., 2012; Zhang et al., 2013).

In this review, we provide a summary of the current understanding of miRNAs and lncRNAs in gliomas with a focus on their roles in GSCs.

## miRNAs IN GSC DIFFERENTIATION

miRNAs are short sequences of 17–25 nucleotides that are not transcribed but have a regulatory function. An RNase III enzyme converts pri-miRNA into pre-miRNA hairpin transcripts that are processed into mature miRNAs and incorporated into a ribonucleoprotein complex called the RNA-induced silencing complex (RISC). The RISC and associated mature miRNA then binds to mRNA and causes a physical block to translation (Ambros and Lee, 2004; Bartel, 2004). Many miRNAs form imperfectly complementary stem-loop structures on the sense strand of the target mRNA. Thus, each miRNA can target multiple mRNA species through recognition of complementary sequences. Upregulation of mature miRNAs may occur as a consequence of transcriptional activation or amplification of the corresponding pre-miRNA locus, whereas downregulation of miRNAs may result from epigenetic silencing or deletion of the corresponding region (Schickel et al., 2008). Although dysregulation of the miRNA-mRNA network has been reported in glioblastoma, little attention has so far been paid to its role in GSCs (Godlewski et al., 2010a). In this section, we describe the information available on the significance of miRNAs in GSCs (**Table 1**).

**Table 1:** List of miRNAs dysregulated in GSCs.

MicroRNAs	Direct targets	Roles in GSC	Reference
miR-17-92 cluster	*CTGF*	Differentiation (-), proliferation (+), apoptosis (-)	Ernst et al. (2010)
miR-451	*CAB39*	Differentiation (-), proliferation (+), apoptosis (-)	Godlewski et al. (2010b)
miR-1275	*CLDN11*	Differentiation (-), proliferation (+)	Katsushima et al. (2012)
miR-138	*CASP3, BLCAP, MXD1*	Differentiation (-), proliferation (+), apoptosis (-)	Chan et al. (2012)
miR-137	*CDK6*	Differentiation (+), proliferation (-)	Silber et al. (2008)
miR-34a	*MET, NOTCH1, NOTCH2, CDK6*	Differentiation (+), proliferation (-), apoptosis (+)	Li et al. (2009b), Guessous et al. (2010)
miR-302-367 cluster	*CXCR4*	Differentiation (+), proliferation (-), invasion (-)	Fareh et al. (2012)
miR-124	*SNAI2*	Differentiation (+), proliferation (-), invasion (-)	Xia et al. (2012)
miR-204	*SOX4, EPHB2*	Differentiation (+), proliferation (-), invasion (-)	Ying et al. (2013)
miR-128	*BMI1, SUZ12*	Differentiation (+), proliferation (-), radiosensitivity (-)	Godlewski et al. (2008), Peruzzi et al. (2013)

*(+) = increased, (–) = decreased.*

### miR-17-92 CLUSTER

The miR-17-92 cluster is thought to be involved in the regulation of GSC differentiation, apoptosis, and proliferation (Ernst et al., 2010). The level of transcripts from miR-17-92 clusters are significantly higher in primary astrocytic tumors than in normal brain tissues and increase significantly with tumor grade progression. A High-level amplification of the miR-17-92 locus has also been found in glioblastoma specimens. Inhibition of miR-17-92 induces apoptosis and decreases cell proliferation in GSCs. mir-17-92 inhibition is also associated with induction of cyclin-dependent kinase inhibitor 1A (*CDKN1A*), E2F transcription factor 1 (*E2F1*), *PTEN,* and connective tissue growth factor (*CTGF*). Of these, the CTGF gene was shown to be a direct target of miR-17-92 in GSCs.

When GSCs are exposed to the differentiation-promoting conditions, downregulation of the oncogenic miR-17-92 cluster is directly related to the concomitant upregulation of CTGF (Ernst et al., 2010).

### miR-124 and miR-137

The initial analysis of miR-124 showed that it promotes neuronal differentiation by targeting the polypyrimidine tract-binding protein 1 (PTBP1) that encodes a global repressor of alternative pre-mRNA splicing; miR reduces the level of PTBP1, which results in an increase in the production of nervous system-specific alternative RNA splicing and promotes the differentiation of progenitor cells to mature neurons (Makeyev et al., 2007). Subsequent analysis showed that both miR-124 and miR-137 are downregulated in high-grade gliomas and up-regulated during adult neural stem cell differentiation (Silber et al., 2008). Transfection of miR-124 or miR-137 inhibits proliferation of GSCs, via suppression of cyclin-dependent protein kinase 6 (*CDK6*), and induces morphological changes in human GSCs and expression of neuronal differentiation markers. Overexpression of miR-124 has consistently been found to inhibit the CD133+ cell subpopulation of the neurosphere and to downregulate stem cell markers, such as *BMI1*, *Nanog*, and *Nestin*. These effects could be rescued by re-expression of *SNAI2*, another direct target of miR-124 (Xia et al., 2012).

### miR-451

Analysis of the miRNA profiles of GSC (CD133+ cells) and non-GSC (CD133– cells) populations showed that several miRNAs, including miR-451, miR-486, and miR-425, are upregulated in CD133– cells. Transfection of cells with miR-451 has been shown to induce disruption of glioblastoma neurospheres (Gal et al., 2008). Interestingly, this study also showed that SMAD proteins, which are associated with GSC regulation, can upregulate miR-451 by binding to its promoter region. Thus, there is a link between miRNAs and well-known stem cell regulating proteins (Piccirillo et al., 2006). Another interesting finding regarding miR-451 is that its expression level is correlated with glucose concentration. High glucose levels are associated with relatively high levels of miR-451 expression, which promote cell growth; miR-451 expression levels decrease under low glucose conditions, resulting in a reduced rate of cell proliferation but an enhanced rate of cell migration and survival in glioblastomas. This miR-451 effect is mediated by liver kinase B1 (*LKB1*). These data indicate that tumor cells can survive under metabolic stress conditions and also seek out locations with more favorable growth conditions by migration influenced through an LKB1/AMPK pathway mediated by miR-451 (Godlewski et al., 2010b).

### miR-34a

miR-34a is tumor-suppressive and is downregulated in human glioma tissues; miR-34a directly inhibits the expression of *c-Met*, *Notch-1,* and *Notch-2* in GSCs (Li et al., 2009b). Notch is a critical regulator of cell-fate during development and also of normal stem cell maintenance (Fan et al., 2006; Shih and Holland, 2006; Fan et al., 2010). Activation of the Notch pathway enhances the stemness, proliferation, and radioresistance of GSCs (Wang et al., 2010). Ectopic expression of miR-34a in glioma cells inhibits cell proliferation, survival, and migration. In addition, miR-34a induces GSC differentiation as evidenced by the decreased expression of stem cell markers and increased expression of differentiation markers (Guessous et al., 2010).

### miR-128

Two studies have described a link between miR-128 and the polycomb repressor complex (PRC). Two major complexes, PRC1 and PRC2, are recognized as key epigenetic regulators during development (Lund and van Lohuizen, 2004) and are required for maintaining self-renewal and multi-potential capability (Richly et al., 2011). The first study demonstrated that miR-128 has a tumor-suppressive function and that this is downregulated in glioblastoma tissue. miR-128 expression significantly reduces glioma cell proliferation both *in vitro* and *in vivo* via downregulation of the oncogene *Bmi-1* that is a component of PRC1. In addition, miR-128 inhibits GSC self-renewal (Godlewski et al., 2008). The second study showed that miR-128 directly targets *SUZ12*, a key component of PRC2. Ectopic expression of miR-128 in GSCs significantly increases their radiosensitivity (Peruzzi et al., 2013). The PRC has been shown to promote normal and cancer stem cell self-renewal and is also implicated in GSC regulation (Abdouh et al., 2009; Suva et al., 2009; Natsume et al., 2013). The findings of these various studies therefore indicate that miR-128 mediates an important epigenetic regulatory pathway in GSCs.

### OTHER miRNAs

Several other miRNAs have been implicated in glioma malignancy. Ectopic expression of the miR-302-367 cluster in GSCs inhibits the CXCR4 pathway resulting in the suppression of stemness signatures, self-renewal, and cell infiltration. Inhibition of the CXCR4 pathway leads to the disruption of the SHH-GLI-NANOG network, which is important for cell self-renewal and tumorigenic properties (Fareh et al., 2012). In both GSCs and non-GSCs, miR-1275 is controlled by a polycomb-mediated silencing mechanism and regulates expression of the oligodendroglial-lineage gene claudin 11 (*CLDN11*). These data illustrate that miR-1275 is regulated by an epigenetic pathway and that it contributes to the phenotypic diversity of glioblastoma tissues. The increased insight into the roles of these miRs may provide a better understanding of basis for the heterogeneity of glioblastomas in the context of human neurodevelopment (Katsushima et al., 2012). Recently, miR-204 was shown to suppress self-renewal, a stem cell characteristic, and the migration of GSCs by targeting the stemness-governing transcriptional factor *SOX4* and the migration-promoting receptor *EphB2* (Ying et al., 2013).

## LncRNAs IN CANCER

Genome-wide studies showed that there are a large number of ncRNAs, including a group termed lncRNAs (Birney et al., 2007). LncRNAs are generally greater than 200 nucleotides and up to 100 kb in length (Mercer et al., 2009). It is known that lncRNAs are mainly transcribed by RNA polymerase II, are polyadenylated and spliced (Wu et al., 2008; Mercer et al., 2009; Ponting et al., 2009). Approximately 15,000 lncRNAs are estimated to occur in human cells and these are frequently expressed in tissue-specific patterns (Derrien et al., 2012). lncRNAs appear to play important roles in a wide range of biological cellular processes including maintenance of stemness, development, and cell survival (Koziol and Rinn, 2010; Zhang et al., 2013). Currently studies detected a set of lncRNAs in each disease using RNA immunoprecipitation with RNA binding proteins coupled with computational approaches.

Long non-coding RNAs are believed to regulate gene expression through four different pathways (Koziol and Rinn, 2010; Hu et al., 2012). First, lncRNAs can bind to chromatin modifying proteins (which have a scaffold function) and recruit these proteins to target loci. These lncRNA complexes can target genes that are closely situated in the genome (*cis-*regulation) or genes that are genomically distant (*trans*-regulation; Nagano et al., 2008; Pandey et al., 2008; Zhao et al., 2008; Gupta et al., 2010; Huarte et al., 2010; Tian et al., 2010; Prensner et al., 2011; Wang et al., 2011). Second, lncRNAs can act as an RNA decoy, that is, they can interact directly with a DNA binding domain to prevent transcription factors interacting with their DNA targets (Kino et al., 2010; Ng et al., 2012). Third, lncRNAs can act as an miRNA sponge, that is, they prevent specific miRNAs from binding to their target mRNAs by competitive binding (Poliseno et al., 2010; Cesana et al., 2011; Karreth et al., 2011). Fourth, lncRNAs can bind to specific combinations of regulatory proteins, such as RNA splicing proteins within ribonucleoprotein complexes (Tripathi et al., 2010; Ng et al., 2012; Schor et al., 2012).

There is increasing evidence to show that a set of lncRNAs is associated with cancer pathogenesis and that these lncRNAs function as regulators in cancer development (Prensner and Chinnaiyan, 2011). lncRNAs that are dysregulated in cancers are listed in **Table 2**. Below, we provide a brief description of some lncRNAs that are associated with glioma tumorigenesis.

**Table 2:** List of lncRNAs dysregulated in cancers.

Name	Cancer type	Biological function	Molecular function	References
**Oncogenic**
*HOTAIR*	Breast, hepatocellular, colorectal, pancreatic, GIST	Promotes invasion and metastasis, modulates cancer epigenome	Scaffold (PRC2, LSD1), guide (*trans*-regulation)	Gupta et al. (2010), Kogo et al. (2011), Yang et al. (2011), Niinuma et al. (2012), Kim et al. (2013)
*ANRIL*	Prostate, leukemia, melanoma	Suppresses senescence via INK4A	Scaffold (PRC1, PRC2), guide (*cis*-regulation)	Pasmant et al. (2007), Yu et al. (2008), Popov and Gil (2010), Pasmant et al. (2011)
*MALAT1*	Lung, prostate, breast, colon, hepatocellular	Regulates alternative splicing of pre-mRNA	Splicing (nuclear paraspeckle)	Ji et al. (2003), Muller-Tidow et al. (2004), Lin et al. (2007), Tano et al. (2010), Tripathi et al. (2010)
*PCAT-1*	Prostate	Promotes cell proliferation, inhibits BRCA2	Scaffold (PRC2), guide (*trans*-regulation)	Prensner et al. (2011)
*CTBP1-AS*	Prostate	Promotes cell proliferation	Scaffold (PSF), guide (*trans*-regulation)	Takayama et al. (2013)
*PCGEM1*	Prostate	Inhibits apoptosis, promotes cell proliferation	Unknown	Srikantan et al. (2000), Petrovics et al. (2004)
*TUC338*	Hepatocellular	Promotes cell proliferation	Unknown	Braconi et al. (2011)
*uc. 73a*	Leukemia, colorectal	Promotes cell proliferation, inhibits apoptosis	Unknown	Calin et al. (2007)
*SPRY4-IT1*	Melanoma	Promotes cell proliferation and invasion, inhibits apoptosis	Unknown	Khaitan et al. (2011)
*ncRAN*	Neuroblastoma, bladder	Promotes cell proliferation and invasion	Unknown	Yu et al. (2009), Zhu et al. (2011)
*PRNCR1*	Prostate	Promotes cell proliferation	Unknown	Chung et al. (2011)
*H19*	Breast, hepatocellular	Promotes cell proliferation, both oncogenic and tumor suppressive functions reported	Unknown	Gabory et al. (2006), Matouk et al. (2007)
**Tumor suppressive**
*GAS5*	Breast	Induces growth arrest and apoptosis	Decoy (glucocorticoid receptor)	Mourtada-Maarabouni et al. (2008), Kino et al. (2010)
*MEG3*	Meningioma, hepatocellular, leukemia, pituitary, gliomas	Mediates p53 signaling, inhibits cell proliferation	Unknown	Zhou et al. (2007,2012), Wang et al. (2012)
*PTENP1*	Prostate, colon	Inhibits cell proliferation	Sponge (PTEN)	Poliseno et al. (2010)
*LincRNA-p21*	Mouse models of lung, sarcoma, lymphoma	Induces apoptosis by repressing p53 targets	Scaffold (hnRNP-k), guide (*trans*-regulation)	Huarte et al. (2010)

### MEG3

Maternally expressed gene 3 (*MEG3*) is a maternally expressed imprinted gene that can also act as an lncRNA. *MEG3* is generally expressed in normal tissues, and its downregulation by aberrant DNA methylation has been found in many types of human cancer (Zhou et al., 2012; Shi et al., 2013). For example, *MEG3* expression in glioma tissues is decreased compared to adjacent normal tissues (Wang et al., 2012). The tumor-suppressive role of *MEG3* is supported by the fact that it can associate with p53 and that this association is required for p53 activation (Lu et al., 2013). Ectopic expression of *MEG3* inhibits cell proliferation and induced cell apoptosis in glioma cell lines (Wang et al., 2012).

### CRNDE

Colorectal neoplasia differentially expressed (*CRNDE*) transcripts are categorized as lncRNAs and have the potential to interact with chromatin-modifying proteins to regulate gene expression through epigenetic changes (Ellis et al., 2012). *CRNDE* is expressed in the fetal brain and in induced pluripotent stem cells; the level of expression increases during neuronal differentiation but no transcripts can be detected in the adult brain (Lin et al., 2011). Intriguingly, *CRNDE* is highly expressed in gliomas. The recent study of Ellis et al. demonstrated a direct interaction between *CRNDE* transcripts and components of PRC2 and the CoREST chromatin-modifying complex. *CRNDE* provides specific functional scaffolds for regulatory complexes, such as PRC2 and CoREST, and may contribute the maintenance of pluripotent state as well as neuronal differentiation (Ellis et al., 2012).

## CONCLUDING REMARKS

Following the discovery of cancer stem cells, it became important to elucidate the mechanisms and the environmental cues that control the differentiation of these cells into the diverse array of cell types that form during tumorigenesis. Epigenetic dysregulation has recently been shown to change the balance between differentiation and self-renewal of cortical progenitor cells and, thereby, to alter the rate and developmental timing of neurogenesis (Pereira et al., 2010). Given that cancer is a disease of faulty cellular differentiation, it is likely that aberrant epigenetic mechanisms involving ncRNAs are involved in glioma tumorigenesis. lncRNAs are increasingly important because of their potential for use in clinical diagnosis and treatment. To date, however, the functions of only a few lncRNAs have been elucidated with respect to tumor biology and there are still many aspects that remain to be resolved. Further investigations are required to clarify the functional roles of lncRNAs in order to elucidate the gene regulatory mechanisms in gliomagenesis. Understanding of the interplays between lncRNAs and genomes, which are reversible alterations, may offer a novel opportunity for the development of molecularly targeted therapies. Nevertheless, a better understanding of the glioblastoma core signaling pathways regulated by ncRNAs and other epigenetic mechanisms will undoubtedly provide novel therapeutic targets and strategies with applications in diagnosis and therapy in glioblastoma.

## Conflict of Interest Statement

The authors declare that the research was conducted in the absence of any commercial or financial relationships that could be construed as a potential conflict of interest.
